# Nasal spindle cell tumor: A case report and literature review

**DOI:** 10.1097/MD.0000000000036833

**Published:** 2024-01-12

**Authors:** Yu Feng, Yunbei Yu, Kai Meng, Maocai Li, Guotao Jia, Yongya Du, Siyu Liu, Lili Gong, Lianqing Li

**Affiliations:** a Department of Otorhinolaryngology Head and Neck Surgery, Liaocheng People’s Hospital, Liaocheng, China; b Shandong First Medical University (Shandong Academy of Medical Sciences), Jinan, China; c Research Department of Liaocheng People’s Hospital, Liaocheng People’s Hospital, Liaocheng, China; d Liaocheng Medical Insurance Fund Audit Center, Liaocheng, China; e Department of Pathology, Liaocheng People’s Hospital, Liaocheng, China; f Department of Otorhinolaryngology Head and Neck Surgery, Liaocheng Dongchangfu People’s Hospital, Liaocheng, China; g Weifang Medical University, Weifang, China.

**Keywords:** benign spindle cell tumor, differential diagnosis, malignant spindle cell tumor, pathological diagnosis, spindle cell tumor

## Abstract

**Background:**

Spindle cell tumors are rare and can occur in any organ or tissue. Due to their rarity the clinicopathological features and diagnostic protocols have not been adequately studied. However, it has become necessary to develop differential diagnosis of spindle cell tumors. Here, we report a case of a nasal spindle cell tumor diagnosed at our hospital in attempt to contribute to this gap in literature.

**Key points from the case:**

A male in his 30s was admitted to our hospital with nasal obstruction that had persisted for several years. Electronic fibrolaryngoscopy revealed a smooth neoplasm within the nasal cavity.

**Main lessons to be learned from this case report:**

The results of this case emphasize that spindle cell tumors have large morphological variations, and it is difficult to determine the origin of tumor cells using hematoxylin and eosin staining alone. Therefore, it is necessary to improve the immunohistochemistry and combine it with clinical symptoms to diagnose the disease.

## 1. Introduction

Spindle cell tumors are rare tumors that were first reported in 1896 by Weiss and Enzinger.^[1]^ They are a group of tumors that are characterized histologically by a mixture of fibroblasts and spindle cells in a matrix of collagen and mucinous material. Additionally, they are rare and can occur in soft tissues, bones, or any part of the human body, such as the epithelial tissues where they manifest as a spindle cell carcinoma (SpCC) or squamous cell carcinoma and in the mesenchymal tissue where they appear as a spindle cell sarcoma or stromal sarcoma. Therefore, their morphological appearance can be either a carcinoma or tumor (carcinomatous or neoplastic). Due to its rarity, the clinicopathological features and diagnostic protocols have not been adequately studied. Direct detection of spindle cell tumor lesions are difficult and require multiple tests, such as immunohistochemical markers. Additionally, clinical, lesion location, and histomorphological characteristics are required to establish a definitive diagnosis. Herein, we report the case of a young man who presented to our hospital with nasal obstruction for several years and was finally diagnosed with a nasal neurofibroma.

## 2. Case report

**Figure 1.**
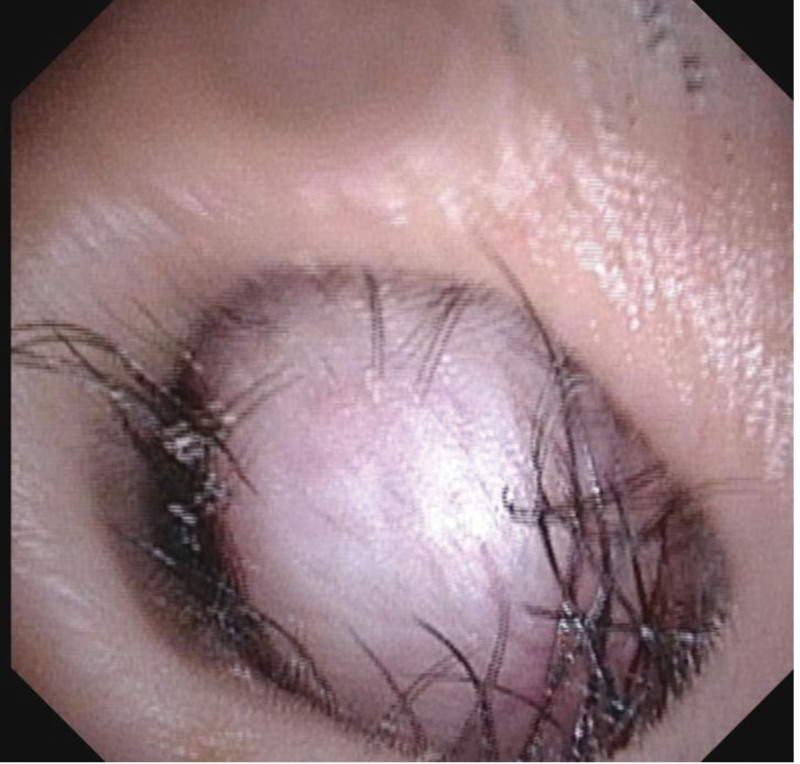
Electronic fibrolaryngoscope revealed a hard white neoplasm in the left anterior nostril with blood vessels on the surface, which could not be viewed inward.

**Figure 2.**
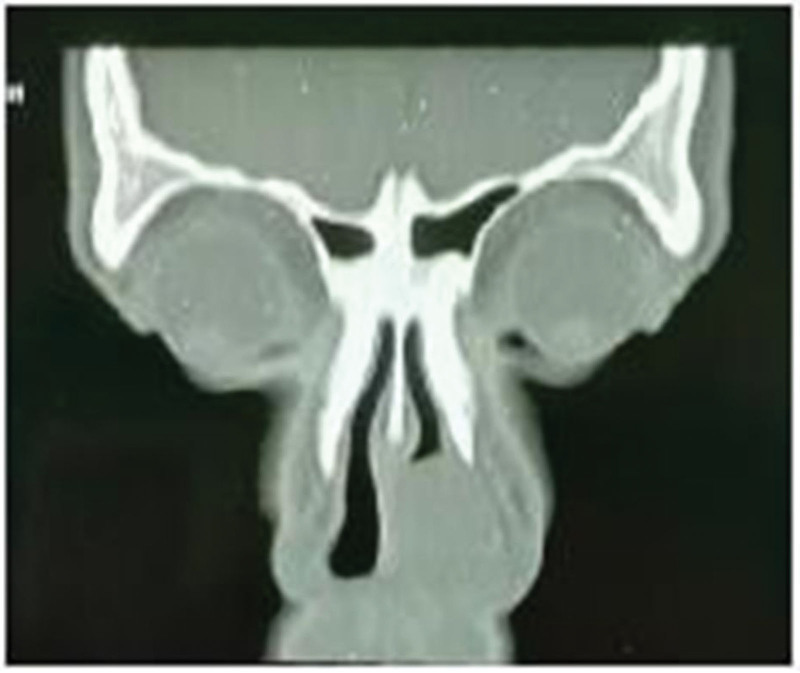
CT of the sinuses showed a left nasal vestibule mass.

**Figure 3.**
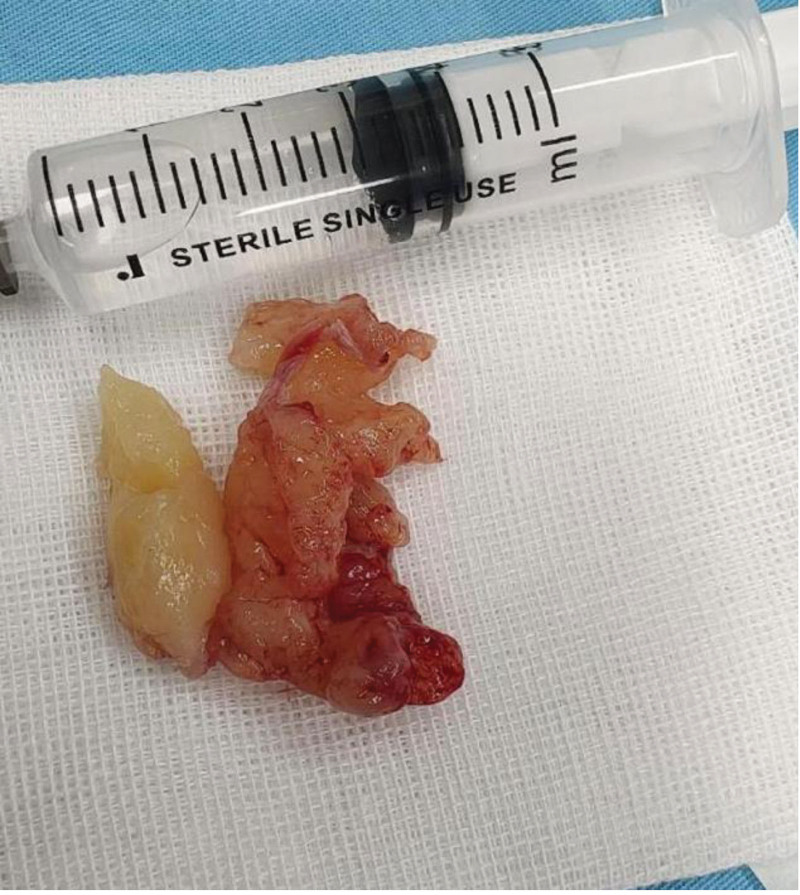
A pile of light red polypoid matter with a volume of approximately 3.5 × 5 cm.

**Figure 4.**
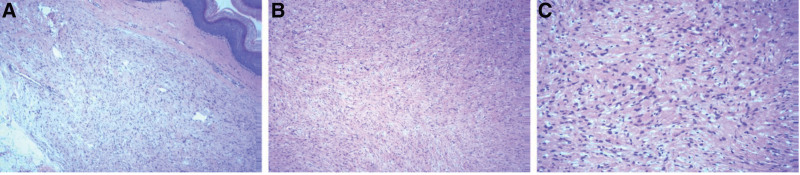
A and B: (hematoxylin and eosin (HE) × 100) tumors were dominated by spindle cells with more obvious atypia and normal reshaped squamous epithelium. C: (HE × 200) Tumor cells showed marked atypia.

**Figure 5.**
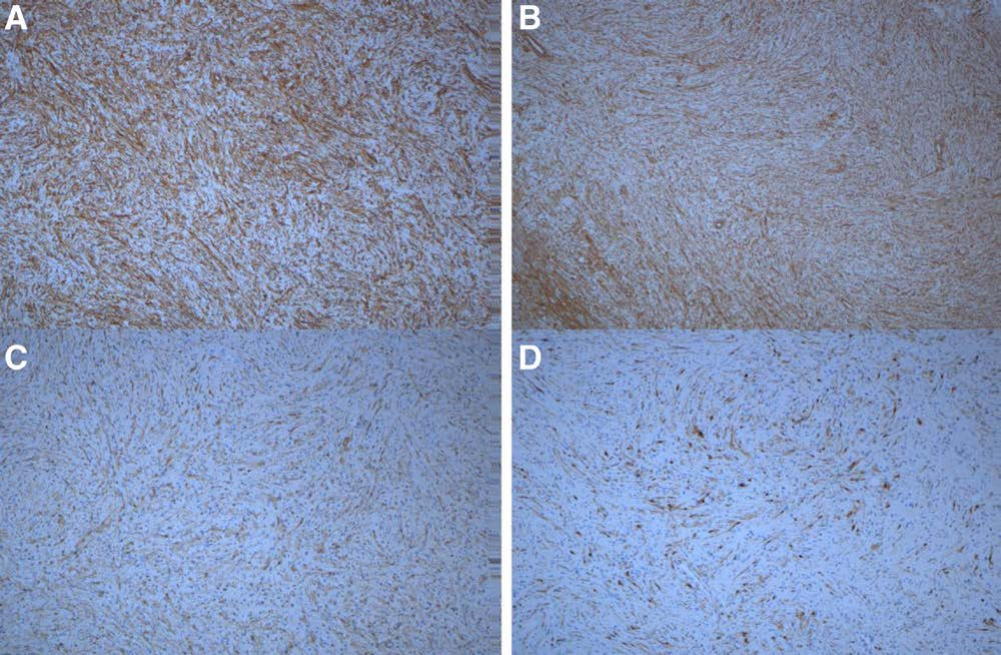
A: Immunohistochemistry: strong positive expression of Vimentin. B: CD99+; C: S-100 was expressed clearly and strongly in atypical cells. D: scattered strong positive expression of cyclinD1.

**Figure 6.**
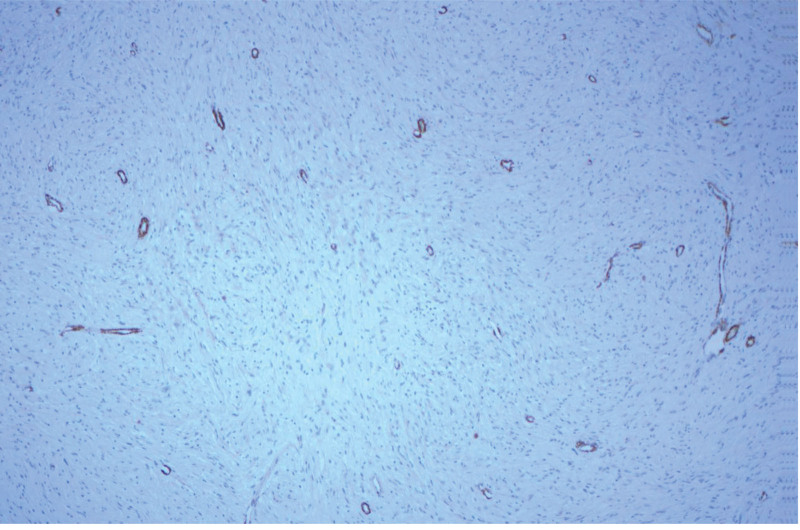
Immunohistochemical SMA showed normal positive expression of spindle tumor cells and interstitial vascular smooth muscle.

A male patient in his 30s was admitted to our hospital because of a left nasal obstruction he had for 8 years prior. The patient accidentally discovered a neoplasm in his left nasal cavity, and in the 8 years the neoplasm gradually grew. At the time of this study, the neoplasm had completely blocked the left nasal cavity, occasionally accompanied by pain and a stench in the nasal cavity. Electronic fibrolaryngoscopy (Fig. 1) revealed a hard white neoplasm on the left anterior nostril, with vessels on its surface that could not be viewed inwardly. Sinus CT (Fig. 2) revealed a left nasal vestibular mass, which was considered a possible cyst. Clinical diagnosis concluded that it was revealed a nasal vestibular cyst. After admission, the patient’s physical examination showed that his general condition was acceptable, and there were no obvious abnormalities in the heart, lungs, or abdomen. Based on the patient’s medical history and related auxiliary examinations, the neoplasm was initially considered a benign. We created a treatment plan and removed the tumor from the left nasal cavity using a nasal endoscope. Surgical findings revealed a mass with a smooth surface and a wide root pedicle in the anterior part of the left nasal cavity, which was located at the lateral third of the nasal threshold, with a size of approximately 2.5 × 2.5 cm. The mass was completely removed (Fig. 3), and no obvious bleeding was observed. Postoperative pathology, hematoxylin and eosin staining (Fig. 4) revealed a spindle cell tumor, consistent with a neurofibroma. To further clarify the nature of the vocal cord neoplasm, immunohistochemical examination was performed, and the results showed (Figs. 5 and 6) the following: Vimentin (+), the BCL-2 (+), CD99 (+), S-100 (+), Cyclin D1 (+), b-catenin pulp (+), SMA (−), STAT6 (−), and CD117 (−). Thereafter, the pathological diagnosis was a benign or intermediate spindle cell tumor. Patient details were not disclosed in this study, and condition articles were reported in accordance with the CARE guidelines.^[2]^

## 3. Discussion

The PubMed database was searched using the keywords “spindle cell tumor” and “head and neck (HN).” Detailed clinicopathological data are presented in Table 1.^[3–8]^ This unique histopathological finding has also been reported in other parts of the body, including the skin and solid organs, in addition to the HN.^[9–11]^Spindle cell tumors are a generalization of tumor cell morphology, and may be benign or malignant. A differential clinical diagnosis of spindle cell tumors is required.

**Table 1:** The PubMed database was searched using the keywords “spindle cell tumor” and “head and neck (HN).”

	Location of the tumor	Benign or malignant	Pathological diagnosis	Treatment
Hartmann S et al(2014)	Sublingual	Malignant	Spindle cell rhabdomyosarcoma	Surgery, radiotherapy and chemotherapy
Mistry P et al (2016)	Sinuses	Malignant	Spindle cell carcinoma	Surgery, radiotherapy and chemotherapy
Yoshida Y et al (2020)	Both sides of the tongue	Malignant	Atypical spindle cell lipomatous tumor	Surgery
Liang Z et al (2021)	Hypopharynx	Benign	Spindle cell lipoma	Surgery
Graja S et al (2022)	The margin of the tongue	Benign	Atypical spindle cells/Polymorphous lipoma (ASCPT)	Surgery
Chang CN et al (2023)	Pituitary gland	Malignant	Spindle cell carcinoma	Surgery

### 3.1. Diagnosis

Spindle cell tumors are mainly composed of spindle cells arranged in fascicles and MATS, mimicking sarcomas, and can occur in epithelial or mesenchymal tissues. In clinical diagnosis, hematoxylin and eosin staining can only provide a preliminary diagnosis between benign and malignant tumors. Therefore, perfecting immunohistochemistry as a tumor source can contribute to further diagnosis.

### 3.2. Differential diagnosis

#### 3.2.1. Benign spindle cell tumor.

##### 3.2.1.1. Head and neck schwannoma.

Schwannoma is a benign tumor derived from Schwann cells of the nerve sheath. The tumor cells were arranged in 2 types, one was AntoniA type (dense type): the tumor cells were spindle-shaped, nuclei were long and round, and the ends were thick and blunt and densely arranged. The other is the AntoniB-type (loose-loose type): the tumor cells are loosely arranged, the cytoplasmic processes are reticular, the cells are stellate, there are many vacuoles or water-like fluids within and between the cells, forming microcapsules or large cystic cavities. S-100 and Leu-7 are positive in immunohistochemistry.^[12,13]^ Clinical signs and symptoms may vary depending on the anatomical location of HN tumors. Most patients present with painless masses, while some may present with pain and tenderness. It can manifest as nasal obstruction or dyspnea when it occurs in the nasal cavity. When it occurs in the pharynx, it manifests as dysphagia. Hoarseness may also occur in the larynx. Neck swelling occurs in the parapharyngeal space.^[14]^

##### 3.2.1.2. Neurofibromas of the head and neck.

Neurofibromas are benign tumors derived from the nerve axis. Typical pathological changes are composed of spindle cells, and the tumor components are mainly hyperplastic glial and Schwann cells.^[15]^ Tumor cells are short spindle, long spindle, or rounded, with uniform nuclear chromatin, nuclei parallel to the cytoplasm, and cells arranged in bundles, longitudinally and vertically. The cells are arranged in bundles and interspersed vertically and horizontally. Neurofibromas of the HN are mostly detected by palpation as a painless mass that gradually increases in size and can be accompanied by dysfunction and mild pain when it compresses neighboring tissues and organs, similar to the clinical manifestation of neurofibromatosis of the HN.

#### 3.2.2. Malignant spindle cell tumor.

##### 3.2.2.1. Low-grade myofibroblastic sarcoma (LGMS).

LGMS is a malignant spindle cell tumor originating from mesenchymal tissues and is mainly composed of myofibroblasts with different degrees of differentiation. Immunohistochemistry is an important method for the diagnosis of LGMS, which is classified into 3 types according to the expression of actin and the nodal protein desmin: actin (+)/desmin (−), actin (−)/desmin (+), actin (+)/desmin (+), and actin (+)/desmin (+). desmin (+).^[16]^

##### 3.2.2.2. Biphenotypic sinonasal sarcoma (BSNS).

BSNS is a group of low-grade spindle cell sarcomas arising in the nasal cavity and sieve sinuses with neurogenic and myogenic differentiation.^[17]^ The clinical presentation of BSNS is nonspecific, with nasal obstruction as the main symptom, nasal obstruction. However, it is poorly demarcated from the surrounding tissues and has infiltrative growth.^[18]^ Tumor cells are spindle-shaped, densely arranged, typically “herringbone” or “herringbone”-like arrangement, with long spindle-shaped nuclei, no obvious heterogeneity, fine chromatin, rare nuclear schizophrenia, and no necrosis. In some cases, antler-like blood vessels are observed in the interstitium. The tumor is characterized by hyperplasia of the overlying respiratory mucosal epithelium, which extends downward or is trapped in spindle cells; in some cases, it is accompanied by rhabdomyoblast-like differentiation. Immunohistochemistry testing is positive for BSNS SMA, calponin, β-catenin, and S-100; and may be focally positive for desmin, EMA, and CK. However, it will be negative for SOX10.^[19]^

##### 3.2.2.3. Spindle cell carcinoma of head and neck (HN SpCC).

HN SpCC is composed of spindle cells of epithelial origin and is a rare variant of squamous cell carcinoma, and is morphologically similar to sarcoma. The tumor is composed of malignant spindle cells with hyperchromatic nuclei.^[20]^ Immunohistochemistry revealed that SpCC was immunopositive for one or more cytokeratins, including AE 1/AE 3, 34 BE 12, and P63. Positive immunohistochemical expression of Cyclin D1 and p16 may indicate an increased risk of local recurrence in patients with SpCC.^[21]^

### 3.3. Treatment

Surgery is the primary treatment for benign spindle and early malignant cell tumors without metastasis. Postoperative adjuvant therapy, such as radiotherapy and chemotherapy, should be performed, according to the pathological and immunohistochemical results, to further reduce the recurrence and metastasis of the tumor so that patients can achieve a longer survival time.

## 4. Conclusions

In this case, spindle cell tumors (prone to benign or intermediate neurofibroma) occurring in the nasal cavity are rare, the differential diagnosis of other benign and malignant spindle cell tumors should be prioritized. Regular follow-up after surgery can help understand the recovery of the patient’s condition and provide corresponding treatment for possible lesions.

## Acknowledgments

I sincerely thank my tutor Li Lianqing for his valuable advice and careful guidance on the revision of the article, and thank all the authors for their selflessly help in data collection.

## Author contributions

**Conceptualization:** Yu Feng, Yunbei Yu, Kai Meng, Yongya Du.

**Resources:** Kai Meng, Maocai Li, Guotao Jia, Siyu Liu.

**Supervision:** Lili Gong, Lianqing Li.

**Validation:** Lianqing Li.

**Writing – original draft:** Yu Feng.

**Writing – review & editing:** Yu Feng, Yunbei Yu.
